# Contested Technologies and Design for Values: The Case of Shale Gas

**DOI:** 10.1007/s11948-015-9685-6

**Published:** 2015-07-25

**Authors:** Marloes Dignum, Aad Correljé, Eefje Cuppen, Udo Pesch, Behnam Taebi

**Affiliations:** 1 Department of Values, Technology and Innovation, Delft University of Technology, Delft, The Netherlands; 2 Department of Policy, Organization, Law and Gaming, Delft University of Technology, Delft, The Netherlands; 3 Belfer Centre for Science and International Affairs, Kennedy School of Government, Harvard University, Cambridge, MA USA

**Keywords:** Public values, Value sensitive design, Responsible innovation, Energy policy, Shale gas

## Abstract

The introduction of new energy technologies may lead to public resistance and contestation. It is often argued that this phenomenon is caused by an inadequate inclusion of relevant public values in the design of technology. In this paper we examine the applicability of the value sensitive design (VSD) approach. While VSD was primarily introduced for incorporating values in *technological design,* our focus in this paper is expanded towards the design of the *institutions* surrounding these technologies, as well as the design of *stakeholder participation*. One important methodological challenge of VSD is to identify the relevant values related to new technological developments. In this paper, we argue that the public debate can form a rich source from which to retrieve the values at stake. To demonstrate this, we have examined the arguments used in the public debate regarding the exploration and exploitation of shale gas in the Netherlands. We identified two important sets of the underlying values, namely *substantive* and *procedural* values. This paper concludes with two key findings. Firstly, contrary to what is often suggested in the literature, both proponents and opponents seem to endorse the same values. Secondly, contestation seems to arise in the precise operationalization of these values among the different stakeholders. In other words, contestation in the Dutch shale gas debate does not arise from *inter*-*value* conflict but rather from *intra*-*value* conflicts. This multi-interpretability should be incorporated in VSD processes.

## Introduction

New technologies may become publicly contested when they fail to take important societal values into account. The acceptability of a technology can be compromised when it (imposes a risk that) negatively affect(s) values that are deemed important by a substantial part of society (Roeser 2011). The framework of value sensitive design (VSD) is geared towards the inclusion of values in technological artefacts and systems. VSD aims to anticipate value conflicts by incorporating societal and ethical aspects in the development of technology (e.g. Friedman et al. 2008). The approach reflects the notion that technologies are not neutral but produce and reproduce values that may be undesirable (Akrich 1992; Ihde 1990; Latour 1992; Oudshoorn et al. 2002; Verbeek 2005; Wolsink 2000; Waelbers 2009; Winner 1980). By identifying relevant public values, and by adequately considering these values in the technological design, the social acceptability of a new technology can be increased, potentially.

Two important prerequisites for embedding values in technological design are, firstly, to identify values at stake and, secondly, to determine which values are conflicting and cannot be accommodated in the same design. A typical example is the design of a car in which at least two key values are embodied, namely safety and sustainability (fuel efficiency). A trade-off between the two values seems to be inevitable; i.e. the lighter the car, the more sustainable it will be in terms of fuel consumption, but the less safe it will become for the driver and the passenger (Van Gorp and Van de Poel 2001). Alternative production methods or the use of other materials could help bypass this conflict to some extent, but eventually a choice needs to be made regarding what level of safety and sustainability we regard as acceptable. Balancing these two values requires addressing the underlying normative question as to which one of the two values is most important, and why.

We argue that values are not only at stake in the design of technologies, but also in the process of their deployment, especially when we deal with major technological projects with significant societal impacts. This means that not only the design of the technological artefact is to be considered, but also the *institutions* in which these artefacts are to be embedded (Correljé et al. 2015; Wüstenhagen et al. 2007). Institutions can be defined as “humanly devised constraints that structure political, economic and social interaction” (North 1991, p. 97). They involve informal institutions, such as taboos, customs, traditions, and codes of conduct, which often inspire formal institutions such as laws, property rights, regulations, and procedures (see also Commons 1931).

Hence, institutions not only ‘prescribe’ technological characteristics and design requirements. They also shape the environment in which the various stakeholders behave and interact and in which social contestations takes place. Participation of stakeholders in policy-making and planning is often suggested as a means to create more democratic and socially supported technologies and projects (Fiorino 1990). Public values are issued in the interaction process between stakeholders with different backgrounds, interests, expectations, and attitudes towards the technology (Walker et al. 2011). This implies that a VSD approach should include stakeholder participation.

This paper focuses on the crucial methodological question that follows from this view on VSD, namely how to identify the relevant and the potentially conflicting values. The relevance of addressing this methodological question has been emphasized earlier (e.g. Manders-Huits 2011; Pesch 2015). This paper takes a first step in answering this question. We argue that the rich public debate is an important source for finding relevant public values. We present an explorative method for a structured identification of public values and their potential conflicts using the data of the public debate. We do this based on a debate regarding shale gas exploration and exploitation in the Netherlands. We believe, however, that the presented methodology has broader applicability for identifying public values at stake in technological and infrastructural projects.

“The Dutch Shale Gas Debate” section of this paper will briefly sketch the Dutch debate on shale gas. “Methods” section presents the method by which we identify the values at stake in this debate. “Values in the Dutch Shale Gas Debate” section presents the values we have found and introduces the difference between *substantive* and *procedural* values. “Discussion” section reflects on these findings in a discussion. In “Conclusion” section we conclude by summarizing the implications of this approach to VSD for science and engineering.

## The Dutch Shale Gas Debate

Shale gas is natural gas which is produced from shale layers deep underground. The Netherlands has a long tradition of natural gas exploration, exploitation and consumption providing much technological and institutional experience in dealing with the societal issues at stake in traditional gas exploitation (Correljé et al. 2003). Anticipating the gradual decline of the Groningen field, the largest European natural gas field, the Dutch government placed renewed emphasis on exploiting smaller fields. Shale gas exploitation is consistent with this policy (Ministry of Economic Affairs 2013a).

When burned, natural gas causes considerably lower CO_2_ emissions, as compared to petroleum and coal. Hence proponents argue that shale gas development, by expanding the gas reserves, has the potential to contribute substantially to a reduction of carbon emissions (Oudeman 2011). Natural gas-fired power plants are then seen as ideal back-up facilities to compensate for the fluctuations in energy supply associated with renewable energy sources like wind and solar.

At the same time, however, there are serious concerns about the negative effects of shale gas exploitation on health, safety and the environment. These involve the risk of leakage of fracking chemicals into drinking water reservoirs, the possibility of earth tremors and local nuisance (e.g. Ministry of Economic Affairs, Agriculture and Innovation 2012). Moreover, it is argued that an expansion of the producible fossil energy reserves with large amounts of previously un-exploitable shale gas will delay the transition to a sustainable energy system. This leads to the paradoxical situation that shale gas is both positioned as a means to enhance welfare and sustainability and as a fuel that obstructs the transition towards a sustainable energy system.

This contested nature became clearly visible in the Dutch shale gas debate. In September 2008, the British company, Cuadrilla Resources Ltd. requested an exploration permit for shale gas in the Dutch province of Noord-Brabant in the South of the Netherlands. Initially, the application proceeded without many difficulties and, by October 2010, the municipalities of Boxtel and Haaren agreed to adjust their zoning plans temporarily to allow for the exploration activities. Yet, when the first permits for exploratory drilling were awarded, local public opinion turned against this initiative. Rather quickly, the local resistance evolved into a broad, nation-wide, anti-shale gas movement, supported by NGOs and several political parties (Metze 2014). There were many important events that led to this resistance. In May 2011, there was an Earthquake in Blackpool (UK) that may have been the result of fracking activities. Additionally, in September 2011, the anti-shale gas film *Gasland* was broadcasted on Dutch national television. This increased the visibility of the film that had already been online since 2009 (Metze 2013). The image of flames from a tap shown in the film and positioned to be related to shale gas extraction had considerable impact (Metze 2014).

The sudden resistance towards shale gas emerged somewhat unexpectedly given the fact that The Netherlands has a long history of easy public acceptance of natural gas exploitation and a tradition of consensus-based policy-making in which industry, public authorities, citizens, and the local population generally manage to agree (Correljé  et al. 2003; Correljé 2011). This makes an analysis of the debate worthwhile both from a methodological and an empirical point of view.

The Dutch government instituted a temporary moratorium and commissioned an investigation of the risks of shale gas exploitation in October 2011. A public consultation, commissioned by the EU in the period between 20 December 2012 and 23 March 2013, indicated a low support of shale gas in The Netherlands. From the Dutch individual respondents, 72 % indicated that no shale gas should be developed in Europe, 17 % indicated that shale gas development should only happen if proper safeguards regarding health and safety are in place, and only 11 % were in favor (Bio Intelligence Services 2013).

In August 2013, the research consortium of three engineering firms concluded that the technological risk would be manageable (Witteveen et al. 2013). This conclusion was communicated to Parliament (Ministry of Economic affairs 2013a). The opposition in Parliament and a number of NGOs heavily criticized the findings of the report, particularly regarding the objectivity and the independence of the engineering firms. Moreover, the report was condemned for being too narrow in focus; excluding the discussion on the need and necessity of shale gas exploitation, and ignoring important environmental effects (Commissie m.e.r. 2013). As this criticism received ample media attention, the study fuelled the controversy rather than resolving it. In September 2013, the government decided to extend the moratorium and commissioned additional investigations regarding location-specific aspects. The results of this study are expected to serve as input for a general framework for decision-making on shale gas exploration (Ministry of Economic Affairs 2013b, 2014a). Until this study is completed at the end of 2015, the moratorium remains in place (Ministry of Economic Affairs 2014b).

## Methods

We observed a rapidly evolving debate extending from the local to the national level, in which the various parties brought in an increasingly wide range of arguments for and against shale gas exploitation. We also observed that in the arguments in the public debate, values play an important role. For example, the EU study indicates the values of health and safety that impact the positioning of stakeholders (Bio Intelligence Services 2013). In addition to these two values, there are also a number of other values at stake; this was vivid in the public debate. Our analysis is based on the arguments that were put forward by various stakeholders in the Dutch shale gas debate. We analyzed key documents from the National Government, NGOs, Industry, and the Dutch Energy Council,^1^ published between May 2010 and November 2013. Drawing upon the sources referred to in these documents, a snowballing procedure was performed which continued until repetition of the arguments indicated that a sufficiently rich overview had been reached. In total, 97 arguments were collected, of which 41 could be identified as being in favor of shale gas exploration and 56 against (see “Appendix” section).

**Fig. 1:**
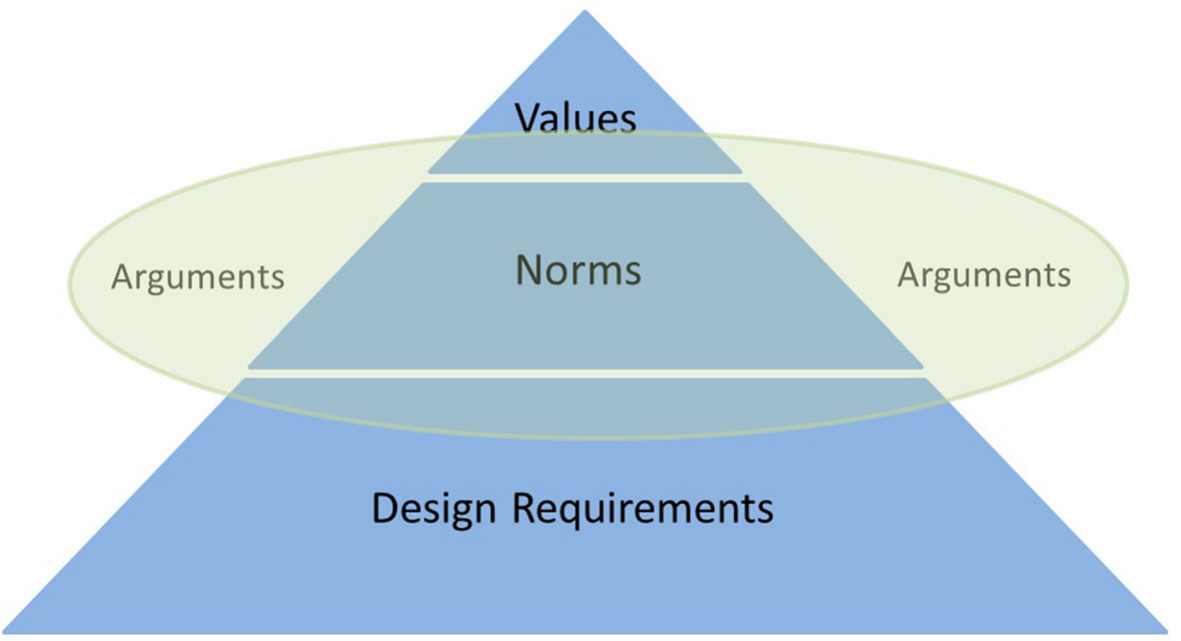
Value hierarchy and arguments (adapted from Van de Poel 2014)

In the next step we analyzed the arguments using Van de Poel’s concept of a “value hierarchy” (Van de Poel 2014). The value hierarchy comprises three levels. At the top, there are a limited number of values, such as safety and resource durability. At the middle level, we find norms that identify actions that support those values. Norms may include *objectives* (such as “maximize safety”, “safeguard the environment” or “minimize costs” without a specific target), *goals* that specify a more tangible target, and/or *constraints* that set boundaries or minimum conditions. At the bottom of the hierarchy, there are design requirements. Design requirements are very specific and detailed and they form the core of (engineering) design (Fig. 1).

We observed that the public debate mainly addressed the mid-level of norms. Norms can be made explicit and expressed in the form of *arguments,* which are put forward in the public debate. Such arguments comprise normative statements about how *the world* should be. From the arguments used in the public debate we gained insight into the values at stake and the associated norms. Figure 1 shows an adapted version of Van de Poel’s value hierarchy, showing the position of the arguments examined in this study.^2^

We then identified the values to which the arguments referred. This was an iterative process. We derived values from the arguments and refined these drawing on the literature on applied ethics and ethics of technology (Friedman and Kahn 2003; Beauchamp and Childress 2009; Taebi and Kloosterman 2014; Friedman 1996; Friedman et al. 2002, 2006; Borning and Muller 2012). This process resulted in a list of 20 values.

If the expressed argument did not clearly refer to a specific value, other documents from the stakeholder were used to determine the precise meaning of that argument taking into account its context. For example, the argument: “Shale gas exploration consumes large quantities of drinking water” is an argument that relates to ‘resource durability’. However, because this argument has also been voiced in the context of the regional drinking water supply and the equal distribution of (the risks of) industrial activities, ‘distributive justice’ was also attributed to this argument. The analysis was validated by cross-verification. The values were coupled to the arguments by one member of the research team, then discussed in a broader group of researchers from the team, until consensus was reached. A second step in the verification involved stakeholders from industry, NGOs, researchers, and policy-makers who participated in expert workshops. In these meetings we presented the definitions of the values to the participants and asked them to link these values to a selection of 47 aggregated arguments. On average 38 arguments were coupled with at least one value similar to our labeling. This provided an indication that our coupling of arguments and values was recognized as valid.

## Values in the Dutch Shale Gas Debate

**Fig. 2:**
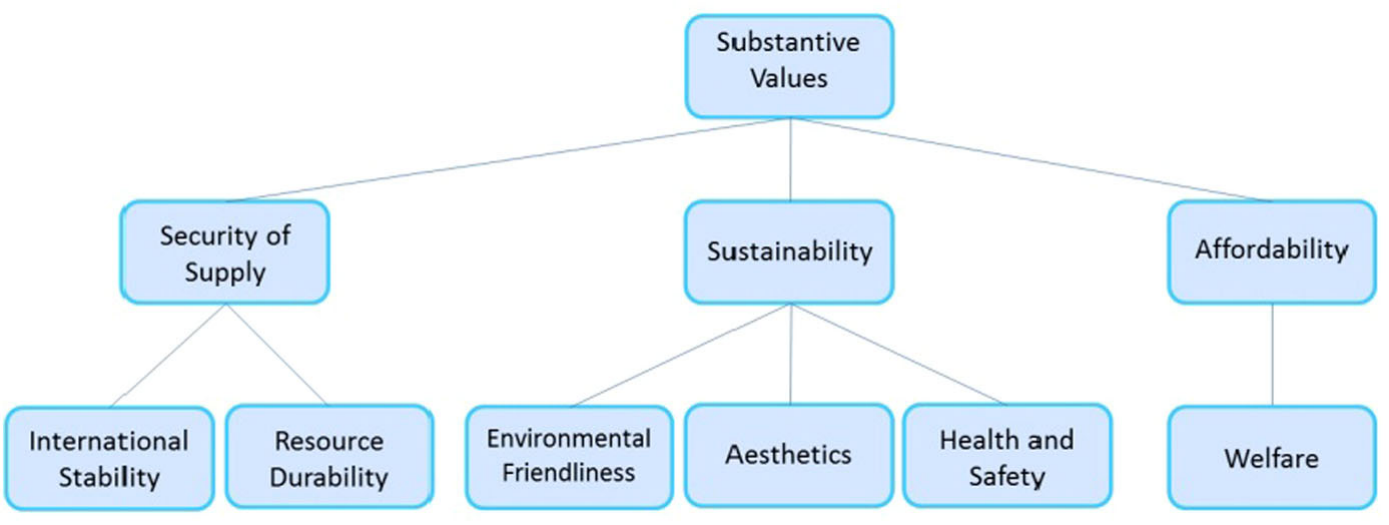
Substantive values in the Dutch shale gas debate

In the analysis of the Dutch shale gas debate we identified two different types of value hierarchies. Firstly, there was a hierarchy of substantive values, or values that relate to the technology of shale gas and the effects of the project(s), which consisted of three main values: sustainability, security of supply, and affordability. Secondly, a hierarchy of procedural values was found. Procedural values relate to the nature of the rules and regulations and the procedures that constitute the decision-making on the exploration and exploitation of shale gas. These latter issues concern aspects such as the limitations of current legislative frameworks, access of stakeholders to the decision-making process, justice and transparency (see Fig. 2; Table 2).

### Substantive Values

The value hierarchy was constructed based on the key documents mentioned earlier. From these documents we specified the three main values of sustainability, security of supply, and affordability. These values refer directly to the three objectives of Dutch energy policy (Ministry of Economic Affairs, Agriculture and Innovation 2011) and EU energy policy [the reduction of GHG emissions, security of supply and the competitiveness of the European economy (European Commissions 2006)].

We then analyzed which norms stem from which values (and likewise, which design criteria stem from which norms). Conversely, we have found norms in these documents, that we have connected to the higher level values (and likewise, design criteria that have been connected to norms).

This process resulted in a list of six *substantive* values, each of which relate to one of the key values of energy policy (see Fig. 2).

Table 1 presents the definitions of each of the substantive values. For example, the argument *“*Shale gas can increase the desired geographical diversity of the gas supply” refers to the value ‘international stability’ and the argument “Shale gas might have adverse environmental impact on flora and fauna” refers to the value of ‘environmental friendliness’.

**Table 1:** Definitions of substantive values and examples of arguments

Substantive values	Definitions
International stability	National and international stability in relation to energy supply, including concerns about import dependence, geopolitical tensions due to changes in energy reserves, and concerns of energy exporting countries regarding demand insecurity
Resource durability	Availability of resources for future generations. This may include the conservation of existing finite resources as well as the development of alternative resources to compensate for depleted resources
Environmental friendliness	“Preserving the status of nature leaving it no worse than we found it” (Taebi and Kadak 2010, p. 1347). This value is presented here in the non-anthropocentric mode, which assigns an inherent value to the environment
Aesthetics	The intrinsic value of the beauty of nature. Changes in the landscape can impact the experienced beauty of the landscape
Health and safety	“[A] state of complete physical, mental and social well-being and not merely the absence of disease or infirmity” (WHO 1948). An argument relates to the value of health when it compromises, or refers to, the state of well-being as defined by the World Health Organization or when it inhibits people from reaching this state
Welfare	Affordability and economic viability of the decision (not) to pursue shale gas exploration and exploitation

Arguments may also refer to two values at the same time. For example, the argument “To realize a carbon extensive economy in 2050, a balance between fossil and renewable energy is required”, links to both ‘resource durability’ and ‘environmental friendliness’. We will further elaborate on this issue below in the discussion section.

Each of the arguments was assessed on the basis of the themes it addresses. This gives an overview of the thematic scope of the values in the debate (Table 2).

**Table 2:** Substantive values and themes in the Dutch shale gas debate

Substantive value	Theme(s)
International stability	Geopolitical stability
Environmental friendliness	Groundwater pollution, water use, impact on flora and fauna and the undesirability of fossil fuel use
Resource durability	Fossil fuels, water
Aesthetics	Landscape impact
Health and safety	Groundwater contamination, nuisance, global warming, and seismicity
Welfare	Costs and benefits

### Procedural Values

Three procedural values were identified: *distributive justice*, *procedural justice*, and *accountability*. Each of these values relate to different aspects of the procedure surrounding shale gas exploration and exploitation (see Table 3 for definitions).

**Table 3:** Definitions of procedural values

Procedural values	Definitions
Accountability	“[S]ound political and legal basis with a corresponding institutional framework” (Flüeler and Blowers 2007, p. 17)
Distributive justice	The fair distribution of costs, benefits, and other positive and negative external effects, including both spatial and temporal distributive justice. The spatial part refers to distribution of negative and positive consequences in a physical spatial sense. The temporal aspect includes intergenerational issues and includes exploitation of resources for future generations, as well as the environment we leave behind
Procedural justice	Transparency, honesty as well as timely, full, and unbiased information in the procedure of planning, exploratory drilling, and exploitation (adapted from Hall et al. 2013)

While these procedural values were present in the debate, the distinction between substantive values and procedural values might not be as clear-cut as suggested in Figs. 1 and 2. The interpretation of a value can also determine whether it is a procedural or a substantive value. For instance, when the value ‘distributive justice’ refers to (questions regarding) *patterns of distribution* of an important good or commodity, ‘distributive justice’ is a procedural value. When it focuses on the *unit of distribution*; i.e. what is the entity or unit of benefit, good or commodity that is to be distributed, it is a substantive value. This later interpretation would, for example, be present in a (hypothetical) discussion regarding the fairness of distribution of two goods with comparable (though not the same) characteristics in which one of the goods is expected to run out. The question that can then arise is whether or not the second good is a fair substitute. In the analysis of the Dutch shale gas debate, ‘distributive justice’ is a procedural value, since we have only observed the procedural interpretation of this value in the debate.

**Fig. 3:**
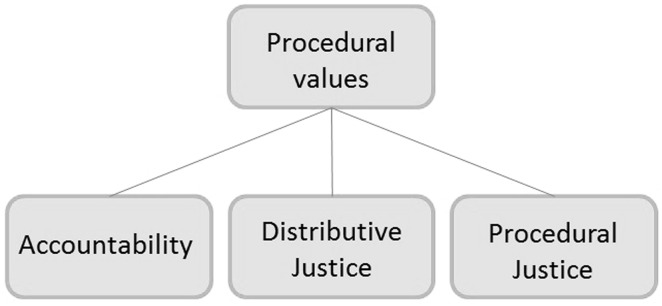
Procedural values in the Dutch shale gas debate

Distributive justice was often used to indicate the (un)fair distribution of costs and benefits among groups and between generations such as the distribution of adverse effects (e.g. pollution or the risks of accidents over time and over locations) or the benefits (e.g. economic gains) (Fig. 3).

Table 3 presents the definitions of each of these values that were used in the debate for linking the values to arguments. For example, the argument “The prospective drilling site of the municipality of Boxtel is located near a railway. It is undesirable to locate two activities with risk together” links to the procedural value of ‘distributive justice’ and the substantive value of ‘health & safety’.

The procedural values were also assessed in terms of their thematic scope (Table 4). The timing theme in Table 4 relates to both the timely disclosure of information and to the timely involvement of stakeholders. These concerns pertain to the desire for recognition and the ability to influence the process of decision-making.

**Table 4:** Procedural values and themes in the Dutch shale gas debate

Procedural values	Theme(s)
Distributive justice	For local populations and future generations
Procedural justice	Timely (formal) participation of stakeholders and the disclosure of relevant information
Accountability	Legal and practical arrangements for safe practice and the allocation of responsibility in case of accidents and incidents including (absence of) trust that arrangements will be followed and would prove to be adequate

## Discussion

Our analysis of the Dutch shale gas debate resulted in two interesting findings. First, we observed that all values, both procedural and substantive, are shared amongst different stakeholder groups, both proponents and opponents. This shared mobilization of similar values holds for both procedural and substantive values. However, this does not mean that all stakeholders agree on the interpretation of the values. For example, an advocate’s supportive argument involving ‘environmental friendliness’ is: “Shale gas offers the opportunity to realize cheap and relatively quick CO_2_ reduction,” whereas an anti-shale gas stakeholder refers to the same value in the following argument: “Shale gas is a fossil fuel and cannot be sustainable.” While the value of environmental friendliness was shared, this shared value also brought up the discussion topic of whether or not some fossil fuels can be more or less environmentally friendly.

Also, procedural values were endorsed by stakeholders. In this case, proponents generally seemed satisfied with the existing institutional frameworks while opponents demanded additional norms and restricting criteria. As regards the value ‘distributive justice’, however, both proponents and opponents identified the need to come up with new norms and criteria for the improvement of current institutional structures. Various arguments criticized the limited possibilities for the compensation of local communities. The issue of compensation was, however, a source of controversy in itself. Compensation is for example being criticized because it can be seen as *bribery* (consistent with e.g. Ter Mors et al. 2012; Hannis and Rawles 2013).

The second observation is that there are many shared values but conflicts arise about how different stakeholders translated or operationalized a shared value. This finding contrasts with empirical studies on cases in which two or more values conflict; e.g. usability and privacy can conflict in software design processes; safety and sustainability seem to oppose each other in car design, and the safest nuclear reactor is not necessarily the most secure one (e.g. Friedman, et al. 2008; Van Gorp 2007; Taebi and Kloosterman 2014; Oosterlaken 2014). What all these cases have in common is that trade-offs between these values are inevitable. Our shale gas study showed that, rather than an *inter*-*value* conflict that necessitates a value trade-off, there seems to be an *intra*-*value* conflict which pertains to different understanding of how a particular value could best be served. This suggests that VSD should be expanded by looking beyond value trade-offs in order to develop a more nuanced understanding of the conflicting operationalization of values and how to manage value conflicts when developing design requirements. Here we would like to point to John Rawls’ distinction between *concept* and *conception*; i.e. while virtually everybody would endorse the notion that we need a *just* society, there are various opinions about of what *justice* exactly entails. Some might argue that justice is understood as the fair distribution of basic goods while others see justice as the respect of fundamental human rights, and again another group considers justice the course of action that maximizes the utility for the greatest number of people. To put it in other words, there seems to be a consensus about the *concept* of justice whereas there are drastically different *conceptions* of justice (Rawls 1971; see also Hart 1961). This theoretical difference has also been observed empirically by Doorn (2012); i.e. while everybody would endorse the notion of responsibility, stakeholders’ rationales for distributing responsibility in an R&D network (or their conceptions of responsibility) could differ. In line with Doorn (2012), we have empirically shown that at the concept-level there is a great degree of consensus regarding the central values that society upholds for the development of a technology. Even in the fierce shale gas debate with all its controversies, this consensus could be found. It is therefore important to understand public values at the level of *conception*, since controversies seem to arise from a different conception, or different operationalization of the same value.

To reveal conceptions we need to understand how a certain value is being operationalized into a norm. For instance, the value of safety in shale gas drilling could relate to groundwater contamination as a result of drilling but it could also relate to the potential pollution from accidents with trucks that supply chemicals for fracking. In order to take such different conceptions of safety into consideration, we have to analyze the public debate. Each of these conceptions will eventually result in different norms and, consequently, different design criteria. Conceptions reveal themselves in the translation steps we make from the top level of value to the mid-level of norms in a value hierarchy (see also Van de Poel forthcoming).

Value sensitive design holds the potential to reconcile value conflicts and thus to lead to more responsible and socially-supported technologies, institutions and procedures. Our results show however, that a consensus on values may appear a fake consensus, since it can hide conflict at the level of norms. This means that value sensitive design needs to account for the divergent and dynamic ways in which a value is translated into different norms, i.e. the divergent conceptions of a value. Value sensitive design will benefit from dialogue between stakeholders, in which the two levels of values and norms are made explicit and the relation between those is jointly explored. This helps stakeholders to reflect on their own (conceptions of) values and relate these to the values of other stakeholders, which will contribute to the mutual understanding of different positions in the debate. Ideally, it leads to shared norms, or shared conceptions, that can overcome previous conflicts.

## Conclusion

VSD has originally only focused on the design of artefacts. We argue that VSD is also relevant for the design of institutions as well as participation procedures (for an elaborate discussion see Correljé et al. 2015; Taebi et al. 2014; Van den Hoven et al. 2014). We focus on one of the methodological challenges of VSD: the identification of values and value conflicts prior to and during the developments of a new technology by drawing on the arguments expressed in the public debate. We identified two hierarchies of values from the debate: substantive and procedural. Both types of values are important to opponents and proponents of shale gas because all stakeholders operationalized the same values (both procedural and substantive) in the debate. Thus we conclude that contestation can be characterized not as *inter*-*value* conflicts, but rather as *intra*-*value* conflicts in which stakeholders interpret the same relevant values differently.

In sum, we argue that VSD needs to start by identifying values associated with contested technologies. One way to do so is by examining the arguments expressed in a public debate and analyzing their underlying values. Insight into stakeholders positions can help in facilitating a timely and iterative discussion about values, norms and design criteria. To create legitimacy, such a process should be open for new information and new insights to allow (re)positioning and enrichment of viewpoints (Pesch 2014). Deliberate design for values is not a process in which the majority rules because minority opinion may include values that are relevant (Taebi et al. 2014; Dunn 2001). In other words, to create and implement a technological design, we must look beyond technology itself and iteratively include institutions and stakeholder interactions to acknowledge the complex and dynamic embedding of a (new) technology in a societal context. Such an approach may not prevent contestation to arise, but it may present a deadlock as a consequence of such contestation.

## Appendix: Arguments and Values Dutch Shale

### Supporting Shale Gas Exploration

1. Shale gas can increase the desired geographical diversity of the gas supply (*international stability*).
2. Shale gas can enhance the security of supply (*international stability, resource durability*).
3. Shale gas can enhance geopolitical stability (*international stability*).
4. The increased gas supply by unconventional exploration such as shale gas, will reduce import dependency (*resource durability, international stability*).
5. Shale gas exploration will increase the share of short term gas contracts and increase the market share of European natural gas, this creates the possibility to spread economic risks (*welfare, international stability*).
6. In contrast to solar and wind energy, shale gas does not have fluctuations in availability (*international stability*).
7. Shale gas can enhance European security because Europe is dependent on Energy import while military investments are simultaneously being reduced (*international stability*).
8. Gas is becoming expensive and scarce, immediate action is required (*welfare, resource durability*).
9. To realize a carbon extensive economy in 2050, a balance between fossil and renewable energy is required (*resource durability, environmental friendliness*).
10. Shale gas is just natural gas and that is an important transitional fuel (*environmental friendliness, health & safety, resource durability*).
11. Shale gas provides the opportunity to develop a sustainable European energy supply using the cleanest available fossil fuel (*international stability, environmental friendliness, resource durability*).
12. Shale gas offers the opportunity to realize cheap and relatively quick CO_2_ reduction (*environmental friendliness, welfare*).
13. Because of shale gas exploration, power plants can shift from using coal to natural gas, reducing coal consumption and CO_2_ emissions (*environmental friendliness*).
14. Drilling through water layers brings about risks. These risks should be considered and adequate exploration and exploitation technology should be used (*health & safety, environmental friendliness*).
15. Shale gas exploration has limited impact on the landscape (the flow of gas is almost silent, there are only some valves and pipes, wired fence, and a temporary drilling construction. Once drilling stops hardly anything is visible) (*environmental friendliness, aesthetics*).
16. Drilling in The Netherlands would happen very deep, reducing the risk of groundwater contamination compared to the USA (*health & safety; environmental friendliness*).
17. The drill and fracking technologies are proven and safe (*health & safety*).
18. The nuisance for the local population is only temporary and will be reduced to a minimum (*health & safety*).
19. Shale gas requires water. However, in the Dutch province of Noord-Brabant shale exploitation would be <1 % of the industrial water use (*environmental friendliness*).
20. The climate footprint of shale gas can be reduced by searching for synergy with geothermal energy (*environmental friendliness*).
21. Exploratory shale gas drilling can also contribute to increasing knowledge on the possibilities of geothermal energy. Chemicals are also required for geothermal energy (*environmental friendliness*).
22. If shale gas exploration is not continued, the dependency on Russian gas imports will increase. Due to the transportation distance this imported gas has a higher climate footprint than local shale gas (*environmental friendliness*).
23. Fracking is not new. There is little difference between fracking in conventional fields and fracking of unconventional gas. Fracking has even been used in the shale oil exploitation in the small gas field near the municipality of Waalwijk (*health & safety*).
24. Drilling through earth layers is not polluting, it is similar to other activities such as groundwater extraction (*environmental friendliness*).
25. If seismic effects were to occur with shale gas exploration, the effects would be a consequence of fracking (and not of additional compression). The tremors can therefore easily be controlled by reducing, or possibly stopping, the fracking activities (*welfare, health & safety*).
26. Seismic effects of shale gas exploration can occur when drilling takes place through existing faults. This is unlikely in The Netherlands since all faults are well-known (*health & safety, welfare*).
27. The decision for sustainable energy is a decision for expensive energy. This can negatively impact the economic position of the EU especially if other regions choose cheap fossil fuels. Shale gas can provide cheaper energy (*welfare*).
28. Shale gas exploration can be very profitable (*welfare*).
29. The Dutch conventional gas resources are depleting. This will impact the state revenues. Shale gas can provide some relief for this depletion (*welfare*).
30. Due to the shale gas revolution, the USA has become an energy paradise. This can attract European energy intensive industry the USA and the EU will lose competitiveness. Shale gas can reduce this effect (*welfare*).
31. The energy intensive chemical industry will not make new investments in Europe if the expected future European gas prices are higher than in other regions (*welfare*).
32. Shale gas fits the experience and positioning of The Netherlands as a leading gas country (*welfare*).
33. Shale gas production can strengthen the economy, provide jobs, and increase possible investments in social security, public policy, safety, and infrastructure (*welfare*).
34. Shale gas can create additional jobs, increase tax revenues, reduce our dependency of imported gas, and increase our balance of payments (*welfare*).
35. If shale gas exploration does not continue, the dependency on Russian gas imports will increase. This will negatively impact the Dutch treasury (*welfare*).
36. Current rules and regulations in The Netherlands are stricter than in the USA and are adequate for the safe extraction of shale gas (*accountability*).
37. Methane leakages in the USA were likely caused by leakages at the drilling sites. It was a combination of bad drilling and bad supervision. In The Netherlands, supervision is much stricter (*health & safety, accountability*).
38. Groundwater pollution is also a risk with conventional gas exploration. However, these risks can be well managed and we have done so for the last 50 years (*health & safety, accountability*).
39. The Dutch and European environmental regulations regarding shale gas exploration are much stricter than those of the USA. This regulation prevents the environmental damage that was endured in the USA (*health & safety, environmental friendliness, accountability*).
40. New regulation should be formulated to allow landowners (or tenants) to share in the profits of shale gas exploration on their land. This can increase the speed of well development (*distributive justice, welfare*).
41. The rules and regulations regarding mining ensure that shale gas exploration will be conducted safely (*health & safety, procedural justice*).

### Opposing Shale Gas Exploration

1. Shale gas is a fossil fuel and cannot be sustainable (*resource durability, environmental friendliness*).
2. Shale gas exploration requires large quantities of water. This can have consequences for the international water (supply) system (*international stability, resource durability*).
3. Shale gas exploration consumes large quantities of drinking water (*resource durability, distributive justice*).
4. Natural gas is seen as an important transitional fuel. Shale gas exploration might threaten support of natural gas in this transition process (N&M) (*health & safety, environmental friendliness, resource durability*).
5. The Dutch province of Noord-Brabant has many geological faults thus increasing the chance of migration of chemicals to the surface (*health & safety, environmental friendliness*).
6. The proper risk assessment of the horizontal drilling should include the surroundings. In the vicinity of the Boxtel/Haaren drilling site there is a kerosene pipeline system as well as a railway network over which hazardous materials are transported (*health & safety, environmental friendliness*).
7. Noise and pollution from trucks that supply materials for fracking will cause discomfort (*environmental friendliness, health & safety*).
8. Shale gas exploration increases the risk of local accidents (such as possible blow outs of wells, road accidents with trucks carrying chemicals and possibly radioactive-water from the drilling site) (*health & safety, environmental friendliness*).
9. The chemicals used in shale gas exploration could, especially around faults, migrate to the surface, causing pollution (*health & safety, environmental friendliness*).
10. Shale gas requires more drilling sites than conventional gas exploration. This increases the chances of leakages and accidents (*health & safety, environmental friendliness*).
11. Shale gas requires more drilling sites than conventional gas exploration. This causes problems after the exploration since it is impossible to permanently seal the wells considering steel and concrete corrosion (*environmental friendliness*).
12. Methane leakage can lead to economic damage due the dead of livestock (*welfare, environmental friendliness*).
13. Shale gas might have adverse environmental impact on flora and fauna (*environmental friendliness*).
14. Soil may become brackish because of movements of salty ground layers due to shale gas exploration and could impact agriculture (*environmental friendliness, welfare*).
15. Shale gas may impact groundwater movements and may otherwise impact the environment based on the use of large quantities of water (*environmental friendliness*).
16. Adding chemicals in water impacts the purity of the water (*environmental friendliness*).
17. Dutch natural gas supplies will last until 2025, there is time to conduct additional research on shale gas and to prevent environmental damage such as in the USA (*environmental friendliness*).
18. Compensating landowners or tenants is bribing and should not be pursued (*distributive justice*).
19. The fracking fluid of shale gas exploration can bring radioactivity from the subsurface above ground (*health & safety, environmental friendliness*).
20. The toxic wastewater from shale gas exploration is often insufficiently purified, causing pollution in sewage systems, rivers, or the drinking water supply (*health & safety, environmental friendliness*).
21. Shale gas can hinder the investment in renewable energy (*environmental friendliness*).
22. Additional gas supply through shale gas exploration reduces the gas price. This may increase the energy consumption and increase greenhouse gas emissions (*environmental friendliness, resource durability, welfare*).
23. A carbon footprint should be made to help decide whether shale gas exploration is desirable (*health & safety, environmental friendliness, welfare*).
24. Shale gas exploration can cause seismic effects that can damage subsurface infrastructures, possibly causing polluted groundwater (*health & safety, environmental friendliness*).
25. The gas price may drop because of shale gas exploration and could negatively impact employment possibilities in the renewable energy sector (*welfare, environmental friendliness*).
26. The long term consequences of the residue chemicals in the ground are unclear (*distributive justice, health & safety, environmental friendliness*).
27. The geological consequences of a fracked layer in the long term are unclear (*distributive justice, health & safety, environmental friendliness*).
28. The large quantities of water that are used for shale gas exploration could negatively impact water availability for agriculture (*welfare, health & safety*).
29. In the province of Flevoland there are windmills with deep foundations. Shale gas exploration might impact these structures (*health & safety, welfare*).
30. The province of Flevoland suffers from a dropping surface. Shale gas might increase this (*health & safety, welfare*).
31. Methane leakage in the home can cause health effects and economic damage (*health & safety, welfare*).
32. Shale gas exploration could result in drinking water pollution (*health & safety*).
33. Methane leakage would increase global warming (*health & safety; environmental friendliness*).
34. The chemicals used with shale gas exploration could leak in the groundwater and enter the drinking water system (*health & safety*).
35. Shale gas exploration in The Netherlands is more expensive than in the USA because of the high population density, stricter legislation, and higher labor costs. There is insufficient attention for the financial risks of government participation through EBN (accountability; welfare).
36. A possible drilling site is near the datacenter of the Rabobank. Seismic effects on this location (caused by shale gas exploration) can disrupt the data transfer (*welfare*).
37. Shale gas does not match the green image that is important to our municipality (*welfare*-*image*).
38. Discussion on shale gas exploration impacts property value (*welfare*).
39. All risks should be included in the price of shale gas to assess whether it is economically viable (*welfare*).
40. The property prices may drop in the vicinity of shale gas exploration sites and it may become more difficult to get a mortgage and/or insurance on such a property (*welfare*).
41. Shale gas is a hype and is not as profitable as it is often portrayed (*welfare*).
42. Internationally, there is decreasing enthusiasm. Several countries banned fracking, have announced a moratorium, or are awaiting for additional research results (*welfare*).
43. Shale gas exploration can cause seismic effects that can damage properties (*health & safety, welfare*).
44. The prospective drilling site in the municipality of Boxtel is located near a railway. It is undesirable to locate two high-risk activities (*distributive justice, health & safety*).
45. Additional legislation regarding shale gas exploration cannot eliminate the risk for pollution due to the migration of pollutants from the subsurface (*accountability, environmental friendliness, health & safety*).
46. There might be conflicting applications of the subsurface. For example, ground that has been exploited for shale gas becomes less suitable as “cap rock” for carbon capture and storage (*resource durability, distributive justice*).
47. The national government can provide permits for drilling in nature reserves; local governments and the public are officially not included in this process (*procedural justice*).
48. On behalf of the Dutch government, EBN participates in gas exploration activities (with a share of 40 %). In the case of shale gas this implies state support of a contested fuel (procedural justice).
49. Shale gas exploration is unsafe because unproven technology will be used and too little is known on shale gas exploration (*accountability, health & safety*).
50. Additional legislation regarding shale gas exploration cannot eliminate the risk for pollution due to seismic effects (*accountability, environmental friendliness, health & safety*).
51. There is limited trust in the companies involved in shale gas exploration with regard to maintaining (safety) standards and integrity (*accountability*).
52. Current procedures do not incorporate the large scale nature of test drilling. The distinction between test drilling and exploration drilling is invalid because the same method, with the same disadvantages, is used. Either test drilling should be subject to environmental legislations such as a MER, or test drilling should focus on the laboratory sample testing (*accountability*).
53. Risks continue after drilling. In the USA there are still risks 15 years after the start of the drilling. Firms may have seized to exist by then (*health & safety, accountability, welfare*).
54. Environmental impact can only be measured properly when a measure prior to exploration is also performed. Currently, this is not in the procedure (*health & safety, environmental friendliness, accountability*).
55. Shale gas exploration requires a lot of infrastructure and transport, this negatively impacts the environment (*aesthetics, environmental friendliness*).
56. Shale gas exploration results in industrial activities in a rural area, this negatively impacts the landscape and surrounding areas (*aesthetics*).

## Footnotes

1 This is a Dutch government advisory council which has a special file on shale gas. This file contained a diversity of shale gas articles and references to reports and enhanced the visibility of these sources.

2 Our modification emphasizes that argument expressed in the debate mostly relate to the level of *norms.* However, there are sometimes arguments that more clearly specify a value and also argument that are very specific and at the level of *design requirements*. Hence, the slight overlap of arguments with the top and lower level in Fig. 1.
